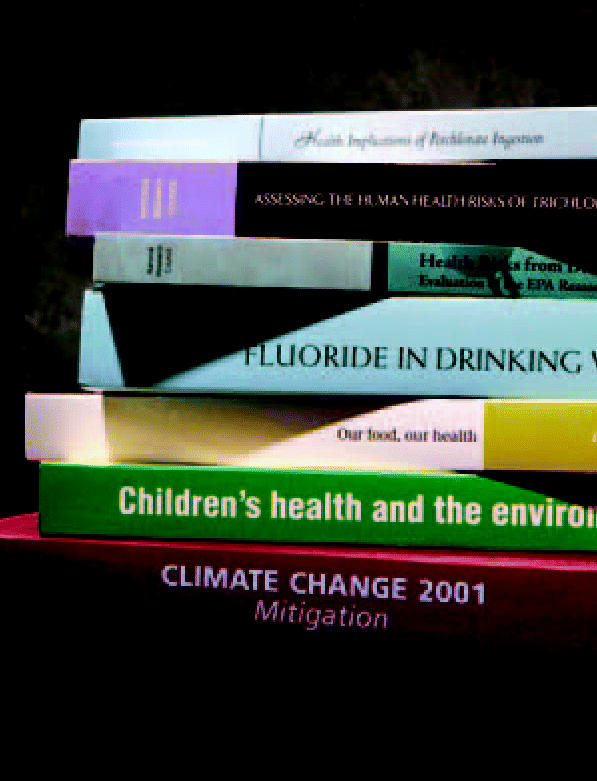# Filling the Translation–Policy Gap

**Published:** 2007-03

**Authors:** Christopher J. Portier, William A. Suk, David A. Schwartz

**Affiliations:** Director, Office of Risk Assessment Research; Director, Center for Risk and Integrated Sciences; Director, NIEHS and NTP, E-mail: david.schwartz@niehs.nih.gov

Formulating health policy without a thorough understanding of the implications of the environment’s influence on health can be compared to building an airplane without an understanding of basic physics: though it may appear to have all the appropriate parts in the appropriate places, without the underlying engineering required for flight, it will never get off the ground. Or worse, it will manage to take off but be unable to sustain itself in flight, with sometimes devastating consequences. Currently, many health policies, including those in the environmental health arena, are formulated and implemented with only a limited foundation in understanding the role of the environment in causing or modulating the disease process. The responsibility can be shared by those in the scientific community who have focused on the science without adequate consideration of its translation, and by public health officials and medical professionals who, perhaps daunted by the technicality of science, have not wholeheartedly sought a complete understanding of the relevant science, or simply haven’t known the right questions to ask. In either case, the result is the same—health decisions don’t always effectively use sound science to guide policy.

The need to fill this gap in improving policy through improved research translation should be a priority for all concerned. The problems of modern society only become more complex over time; similarly, the science required to address these problems, particularly in the area of human health and disease, is increasingly complex. A response to this critical need might be found in a bi-directional approach that trains scientists to effectively translate technical research into understandable components useful to regulators, medical professionals, and public health officials, who, in turn, may use their better understanding of science to direct the scientific community toward the most needed and valuable research. The eventual goal of a program in health policy training is to improve the overall utility of scientific discoveries in environmental health to local, state, national, and international health policies.

The scientific community would benefit from a training program to provide those from an applied, basic, or clinical research background with the knowledge and skills to participate effectively in the larger context of environmental health policy. Examples of such knowledge and skills would include the abilities to estimate health risks from environmental exposures; use environmental factors to define and modify clinical management of a disease; use emerging science to identify and quantify uncertainty in avenues for mitigating health risks in both the clinical arena and the population health arena; use risk assessment to set priorities for managing environmental health hazards; evaluate the impact of environmental exposures on disease etiology; enhance capacity to assess new biomedical technologies for their application to environmental health problems; and use knowledge of the internal structure and function of government, academic, corporate, and advocacy institutions to provide scientific guidance in effecting health policy.

The scientific community would benefit from a training program to provide those from an applied, basic, or clinical research background with the knowledge and skills to participate effectively in the larger context of environmental health policy.

Health policy experts at institutions such as research-intensive universities, hospitals, managed health care systems, health advocacy groups, corporate medical departments, health and environmental consulting firms, state and local health departments, legislative committees, federal regulatory agencies, and international agencies should consider investing time and effort to better understand the scientific community, and to invent new mechanisms to interface science with policy decisions. They should also be prepared to apprise the scientific community of gaps in knowledge that affect the development and delivery of effective policies to help guide the allocation of research resources.

Creation of a training program that encourages scientists to actively participate in health policy discussions by enabling them to more effectively bring what they know to the table would yield multiple benefits. It would provide scientists with a means of quantifying the applicability of their research and guide their future efforts. It would greatly enhance the scientific foundation of public health policy. Such a program would create a cadre of scientists who fully comprehend how health policy decision making can be better informed using information from research in basic, applied, and clinical sciences, and who can utilize the tools available in both environmental health science and the science of health policy to further decision making with regard to environmentally related diseases and health practices.

By focusing knowledge, efforts, and resources on solving environmental health and human disease problems, environmental health policy training would allow public health to soar.

## Figures and Tables

**Figure:**